# Indapamide Increases IRS1 Expression and Modifies Adiponectin/NLRP3/PPARγ Crosstalk in Type 2 Diabetic Rats

**DOI:** 10.3390/antiox11040691

**Published:** 2022-03-31

**Authors:** Mahmoud M. Samaha, Manar G. Helal, Mohamed El-Sherbiny, Eman Said, Hatem A. Salem

**Affiliations:** 1 Department of Pharmacology and Toxicology, Faculty of Pharmacy, Mansoura University, Mansoura 35516, Egypt; msamaha@mans.edu.eg (M.M.S.); manargamal@mans.edu.eg (M.G.H.); hatemsalem57@mans.edu.eg (H.A.S.); 2 Department of Basic Medical Sciences, College of Medicine, AlMaarefa University, Riyadh P.O. Box 71666, Saudi Arabia; msharbini@mcst.edu.sa; 3 Department of Anatomy, Faculty of Medicine, Mansoura University, Mansoura 35516, Egypt; 4 Faculty of Pharmacy, New Mansoura University, New Mansoura 7723730, Egypt

**Keywords:** canagliflozin, indapamide, adiponectin, NLRP3, PPARγ, IRS1

## Abstract

The current study aimed to evaluate the anti-diabetic effects of canagliflozin (CANA) and indapamide (INDA) and their impacts as adiponectin modulators in experimentally induced type 2 diabetes mellitus (T2DM). T2DM was associated with a significant rise in blood glucose level and HbA1C%, andreduced adiponectin and insulin secretions. Moreover, the malondialdehyde (MDA) contents in both the epididymal adipocytes and soleus muscle significantly escalated, while the total antioxidant capacity (TAC) and epididymal adipocyte Nrf2 expression significantly declined. Moreover, serum TNF-α, epididymal adipocyte’s NOD-like receptor protein 3, NLRP3, NF-κB and CD68 expressions markedly escalated, and serum IL-10 significantly declined. Furthermore, there was a significant escalation in PPARγ expression in epididymal adipocytes, with a significant reduction in soleus muscle’s expression of IRS1. CANA and INDA treatments markedly reduced blood glucose levels, increased adiponectin and insulin secretion, enhanced anti-oxidant defenses, and reduced oxidative burden, with marked anti-inflammatory impact. Interestingly, the impact of indapamide on DM indices and oxidative and inflammatory changes was comparable to that of canagliflozin. Nevertheless, indapamide had a superior effect compared to canagliflozin on HbA1c%, expression of IRS1 and reduction of NF-κB and CD68 expressions. INDA could be effective in regulating T2DM, with underlined anti-diabetic, antioxidant, and anti-inflammatory properties. INDA increased IRS1 expression and modified adiponectin/NLRP3/PPARγ crosstalk. The impacts of INDA are comparable to those of the standard anti-diabetic drug CANA.

## 1. Introduction

Diabetes mellitus (DM) is a metabolic syndrome highlighted mainly by both chronic hyperglycemia and abnormalities in fat and protein metabolism due to either the inadequate secretion or impaired action of insulin [1]. Actually, hyperglycemia is considered a significant contributor to the strong oxidative stress usually associated with DM [2]. The increased intake of both glucose and macronutrients is usually associated with the incidence of obesity, another major contributor to type 2 diabetes mellitus (T2DM). Obesity is usually associated with a pro-inflammatory state, enhanced oxidative stress, which interferes with the anti-inflammatory properties of insulin. Moreover, the established insulin resistance, hyperglycemic state, and oxidative stress caused by T2DM induce the development of several diabetic complications, including nephropathy, retinopathy, neuropathy, cardiovascular diseases, and nonalcoholic fatty liver disease (NAFLD) [3].

Various prospective investigations in different ethnic groups have reported low serum levels of adiponectin to be an independent risk factor for T2DM and its associated cardiovascular complications [4]. Along these lines, animal studies have also confirmed adiponectin to be an insulin-sensitizing hormone with anti-diabetic activity [5]. Adiponectin knock-out mice were more prone to developing high-fat diet (HFD)-induced insulin resistance [6].

In skeletal muscle, adiponectin induces fatty acid β-oxidation, with a subsequent decrease in lipid accumulation and an increase in insulin sensitivity [7]. In addition to its insulin-sensitizing properties, adiponectin has been reported to protect against various obesity-related pathologies, including hypertension, heart failure, atherosclerosis, steatohepatitis, airway inflammation, and breast cancer [8].

The number of anti-diabetic drugs available that aim to control adequate blood sugar levels, thus controlling T2DM, is increasing. However, most of these medications have limited efficacy, and their use is usually associated with the development of many undesirable adverse effects, including resistance, weight gain, dropsy, and increased rates of secondary treatment failure [9].

The sodium-dependent glucose cotransporters SGLT1 and SGLT2 are of significant importance for the regulation of glucose homeostasis. They regulate the absorbance of glucose from the diet in the small intestine (via SGLT1) by reabsorbing the filtered glucose in the tubular system of the kidney (primarily via SGLT2 and to lesser extent via SGLT1). Then, glucose is returned into the blood stream, thus inhibiting urinary glucose loss. Currently, three SGLT2 inhibitors are available—canagliflozin (CANA), empagliflozin, and dapagliflozin—and these are now widely approved antihyperglycemic therapies due to their unique glucosuric mechanism. These agents thus represent a new class of oral anti-diabetic agents with a novel mechanism. They have been reported to improve glycemic control and to promote weight loss and blood pressure reduction in patients with T2DM [10].

Indapamide (INDA) is a thiazide-like diuretic with antihypertensive properties. INDA exhibits protective effects against target organ damage in animal models of hypertension. It reduces left ventricle hypertrophy in spontaneously hypertensive rats, and limits the expression of ventricular fibronectin, thus preventing the development of cardiac fibrosis. INDA also hinders nephrosclerosis via the induction of endothelium-dependent vasodilatation. Moreover, INDA exhibits some potentially important pharmacological properties, including blocking voltage-operated calcium channels and promoting the production of prostacyclin, thus limiting sympathetic nerve activation [11]. Furthermore, INDA significantly lowers blood pressure in hypertensive patients with T2DM. Additionally, it preserves patients’ lipid profiles and glucose metabolism, and exerts no potential side effects [12].

The current study was conducted to evaluate the modulatory impact on adiponectin of both CANA and INDA, as well as their subsequent impact on insulin resistance, overall glycemic control, changes in adipose tissue associated with T2DM, and glucose use in the skeletal muscles.

## 2. Materials and Methods

### 2.1. Experimental Animals

Thirty-six adult male Sprague-Dawley rats (240–300 g) were purchased from the Urology and Nephrology Center, Mansoura, Egypt. The rats were maintained 6 rats/cage at 25 °C with a 12 h light–dark cycle until the start of the experimental procedure with access to water and food ad libitum. The Research Ethics Committee of the Faculty of Pharmacy, Mansoura University, Egypt, accepted the experimental protocol and procedures.

### 2.2. Drugs and Chemicals

**Streptozotocin (STZ)** was obtained from Sigma-Aldrich (St. Louis, MO, USA) and was prepared by dissolution in a pH 4.5 citrate buffer immediately prior to use [13]. **Fructose** was obtained from El-Goumhouria Co. (Cairo, Egypt). CANA was received as Invokana^®^ tablets, manufactured by Janssen Pharmaceuticals (Beerse, Belgium), and prepared as a suspension in 0.5% carboxymethylcellulose (CMC) for oral administration. Other excipients in the tablets included hydroxypropyl cellulose, croscarmellose sodium, magnesium stearate, microcrystalline cellulose, lactose anhydrous, iron oxide yellow, polyethylene glycol, polyvinyl alcohol, talc, and titanium dioxide. **INDA** was obtained as DIUREX^®^ tablets (AMRIYA, Alexandria, Egypt), and was prepared as a suspension in 0.5% CMC for oral administration. Other excipients in the tablets included spray-dried lactose, microcrystalline cellulose, croscarmellose sodium (type A), magnesium stearate, Hypromellose, titanium dioxide, and macrogol 400.

### 2.3. Experimental Protocol

The rats were allocated into 6 experimental groups, with 6 rats/group. One group of rats was fed regular standard chow (RSC) for 8 weeks and served as a **normal CTRL**. A further 2 groups were also maintained on RSC for 8 weeks, and received daily CANA (10 mg/kg, orally) or INDA (10 mg/kg, orally), respectively, starting from week 5, and served as **CANA CTRL and INDA CTRL**, respectively. The remaining rats received HFD + 25% fructose in drinking water for 4 weeks, after which the HFD-fed rats were fasted for 12 h, and received a single intraperitoneal injection of STZ (35 mg/kg). The composition of the HFD was cholesterol 2.5%, lard fat 10%, sucrose 20%, and powdered RSC 67.5% [14], with 25% fructose in drinking water [15]. After STZ injection, the glucose was monitored and recorded for 3 successive days in blood samples from the tail vein tip using a blood glucose meter to ensure the incidence of DM.

Diabetic rats that developed DM, confirmed on the basis of recorded blood glucose levels (≥200 mg/dL), were further allocated into 3 experimental groups in a random order. The experimental groups included: **diabetic CTRL**—rats given 0.2 mL of (0.5% CMC, orally) for 4 weeks; **diabetic/CANA** group—rats given oral daily CANA (10 mg/kg); and **diabetic/INDA** group—rats given oral daily INDA (10 mg/kg). CANA and INDA treatments were initiated from week 5 to week 8. Meanwhile, the diabetic CTRL, diabetic/CANA and diabetic/INDA groups were supplied with HFD plus 25% fructose in their drinking water. The overall experimental period was 8 weeks.

The changes in blood glucose and B.W. were recorded twice weekly. The animals were sacrificed by overdose of thiopental sodium (40 mg/kg) at the end of week 8. Post exsanguination, the epididymal adipocytes, the soleus skeletal muscles, and the pancreas were harvested and further processed after washing in ice cold saline.

### 2.4. Determination of Glycosylated Hemoglobin (HbA1c) in the Blood, Serum Total Antioxidant Capacity (TAC), and Insulin, Adiponectin, Tumor Necrosis Factor-α (TNF-α) and Interleukin-10 (IL-10) Concentrations

Blood samples were drawn from the retro-orbital plexus. First, whole blood samples were used for HbA1c determination using rat HbA1c assay kit (Biodiagnostic, Giza, Egypt). Another group of samples were left to coagulate at room temperature and were centrifuged for 15 min at 4000 rpm to separate the sera. These were used for TAC estimation using the Biodiagnostic Kit (Egypt). Additionally, using Cusabio rat ELISA kits (Houston, TX, USA), insulin (Cat. no. CSB-E05070r), adiponectin (Cat. no. CSB-E07271r), TNF-α (Cat. no. CSB-E11987r) and IL-10 (Cat. no. CSB-E04595r) were quantified in serum. All the procedures specified by the manufacturer were followed in all cases.

### 2.5. Tissue Homogenate Preparation and Determination of Malendiof Contents of Malondialdehyde (MDA), Nucleotide-Binding Oligomerization Domain (NOD)-Like Receptor Protein, Nuclear Factor Erythroid 2-Related Factor 2 (Nrf2) and 3 (NLRP3) Epididymal Adipocytes and Soleus Muscle, and Insulin Receptor Substrate 1 (IRS1) Content in the Soleus Muscle

The tissue homogenates were processed according to Samaha et al., 2020 [16]. Using the Biodiagnostic Kit (Egypt), the supernatants of both tissues were used to estimate the contents of MDA in the tissues. Moreover, using Cusabio ELISA kits (USA), NLRP3 (Cat. no. CSB-EL015871MO) and Nrf2 (Cat. no. CSB-E16188m) were quantified in the supernatant of the adipose tissue and the soleus muscle supernatant was used for the determination of IRS1 (Cat. no. CSB-PA047827) according to the enclosed pamphlet from the manufacturer.

### 2.6. Western Blot Analysis

The epididymal adipocytes samples were segmented and suspended at 4 °C in lysis buffer, iced for 10 min, then vortexed for 10 s and centrifuged at 14,000× *g* for 10 min to separate the cell pellets, which were then resuspended in a mixture of cold RIPA buffer (pH 7.4) and 1:300 phosphatase and protease inhibitors Sigma-Aldrich (St. Louis, MO, USA), and incubated on ice for 20 min and recentrifuged at 14,000× *g* for 10 min at 4 °C for removal of cellular debris, and the supernatant was separated to be used for Western blot analysis. The total protein concentration in the samples was determined colorimetrically using the Bradford method. Equal amounts (25 μg) of protein samples were mixed and boiled with SDS loading buffer for 5 min, allowed to cool on ice for 7 min, and then loaded into SDS-polyacrylamide gel and separated using Cleaver electrophoresis, before being transferred onto polyvinylidene fluoride membranes for 30 min at 2.5 A and 25 V for 30 min. Then, 5% nonfat dry milk in TBS-T was used for two hours at 37 °C to block the membrane, and it was incubated overnight at 4 °C with primary antibodies against Nrf2 (Cat. no. ab92946, abcam, USA), PPAR-γ (Cat. no. SC-271392, Santa Cruz Biotechnology, Dallas, TX, USA) and β-actin (Cat. no. SC-69879, Santa Cruz Biotechnology, Dallas, TX, USA) proteins. The blots were then washed three times with TBS-T and incubated with horseradish peroxidase-linked secondary antibodies (Dako) for another 60 min at room temperature and washed three times with TBS-T. The chemiluminescent Western ECL substrate (Perkin Elmer, Waltham, MA, USA) was applied to the blot as instructed, and the chemiluminescent signals were captured with a Chemi Doc imager (Biorad, Hercules, CA, USA), the band intensities were measured and normalized to β-actin [17,18].

### 2.7. Histological and Immunohistochemical Analysis

The epididymal adipose tissue and the pancreatic head were fixed in 10% buffered formalin and processed for stained with hematoxylin and eosin (H&E) stain. The epididymal adipose tissue was also investigated for the expression of both of the nuclear factor kappa B (NF-κB) and the cluster of differentiation CD68 (CD68). Representative sections were photographed (Olympus^®^ digital camera installed on Olympus^®^ microscope, using 400× objective) and analyzed using ImageJ software, with 6 reads for adipose tissue, and then the mean for each rat was determined. The images were further analyzed using VideoTest Morphology^®^ software (Russia) with a specific built-in routine for area, % area measurement and object counting.

### 2.8. Homeostasis Model Assessment of β-Cell Function (HOMA-β)

Homeostasis model assessment β-cell function (HOMA-β) index was determined in accordance with Matthews et al., 1985 [19].

(1) $$\mathrm{HOMA}-\beta =\frac{20\times fastinginsulin\;(\frac{mU}{L})}{\left[fastingglucose\;(\frac{mmol}{L})-3.5\right]}$$

### 2.9. Statistical Analyses

Data represent the mean ± standard error of mean (S.E.M). One-way analysis of variance (ANOVA) followed by post hoc Tukey–Kramer test was used for statistical comparison between the mean values. GraphPad Instat 3.05 and GraphPad Prism 7 (San Diego, CA, USA) software packages were used for statistical evaluations and plotting of graphs. The accepted level of significance was *p* < 0.05.

## 3. Results

Subjecting the drug control (CTRL) group to CANA and INDA treatments induced a non-significant difference compared to the to normal CTRL; thus, these three groups are hereafter collectively referred to as normal CTRL.

### 3.1. Impact of Daily CANA and INDA Treatment on Body Weight

**Figure 1:**
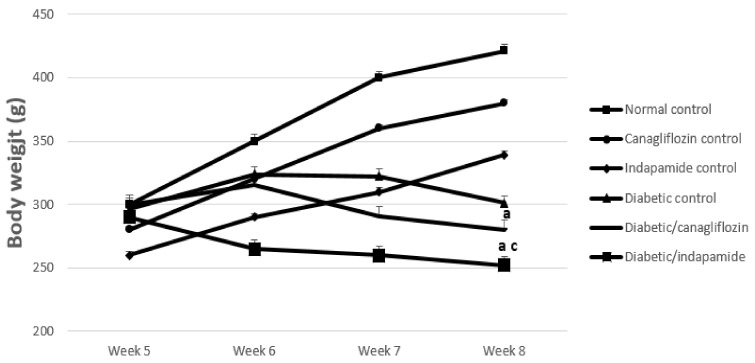
Impact of daily CANA and INDA treatment on B.W.: oral CANA and INDA treatments were initiated once daily for 4 weeks. Data represent the mean ± S.E.M. (6 rats/group). ANOVA followed by post hoc Tukey–Kramer test at (*p* < 0.05) was used for statistical comparison; ^a^ against normal CTRL, ^c^ against initial B.W.

Diabetic CTRL rats demonstrated a significant decrease in B.W. of approximately 29% (*p* < 0.05) compared to the normal CTRL. Daily INDA (10 mg/kg) treatment for 4 weeks resulted in a significant reduction in B.W. of about 16% (*p* < 0.05) compared to the diabetic CTRL, while daily oral CANA (10 mg/kg) treatment for 4 weeks induced a non-significant decrease in B.W. compared to that in the diabetic CTRL (Figure 1).

### 3.2. Impact of Daily CANA and INDA Treatment on Serum Blood Glucose

**Figure 2:**
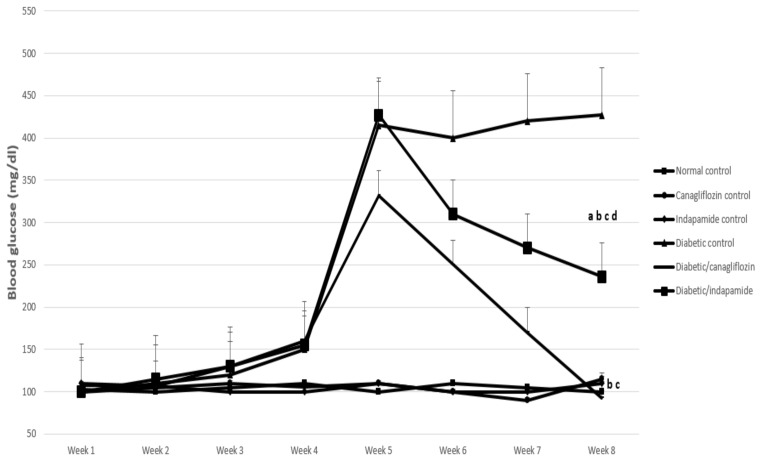
Impact of daily CANA and INDA treatment on blood glucose: oral CANA and INDA treatments were initiated once daily for 4 weeks. Data represent the mean ± S.E.M. (6 rats/group). ANOVA followed by post hoc Tukey–Kramer test at (*p* < 0.05) was used for statistical comparison; ^a^ against normal CTRL, ^b^ against diabetic CTRL, ^c^ against initial blood glucose, ^d^ against diabetic/CANA.

Four weeks post DM induction, blood glucose exhibited a significant increase of about 4-fold (*p* < 0.05) in the diabetic CTRL compared with the normal CTRL. Oral CANA (10 mg/kg) and INDA (10 mg/kg) treatment for 4 weeks significantly reduced blood glucose by about 78% (*p* < 0.05) and 45% (*p* < 0.05), respectively, compared with the diabetic CTRL. Meanwhile, serum glucose significantly decreased by about 72% (*p* < 0.05) and 45% (*p* < 0.05) upon treatment with CANA (10 mg/kg) and INDA (10 mg/kg), respectively, with respect to their former blood glucose levels, but without any significant difference compared to the normal CTRL. CANA (10 mg/kg) induced a superior effect in terms of blood glucose level reduction compared to that of INDA (Figure 2).

### 3.3. Impact of Daily CANA and INDA Treatment on HbA1c%

**Figure 3:**
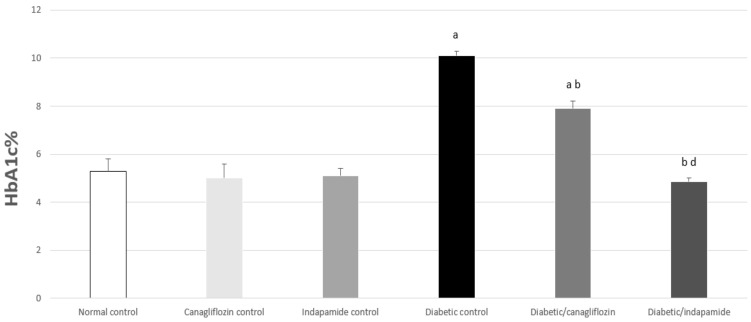
Impact of daily oral CANA and INDA treatment on HbA1c%: oral CANA and INDA treatments were initiated once daily for 4 weeks. Data represent the mean ± S.E.M. (6 rats/group). ANOVA followed by post hoc Tukey–Kramer test at (*p* < 0.05) was used for statistical comparison; ^a^ against normal CTRL, ^b^ against diabetic CTRL, ^d^ against diabetic/CANA.

HbA1c% exhibited a significant increase of approximately 1.9-fold (*p* < 0.05) in the diabetic CTRL compared to the normal CTRL. Oral CANA (10 mg/kg) and INDA (10 mg/kg) treatment for 4 weeks caused a significant decrease of about 22% (*p* < 0.05) and 52% (*p* < 0.05), respectively, in HbA1c% compared to the diabetic CTRL, but without any significant difference compared to the normal CTRL. INDA (10 mg/kg) reduced HbA1c% more effectively than CANA (Figure 3).

### 3.4. Impact of Daily CANA and INDA Treatment on Serum Insulin

**Figure 4:**
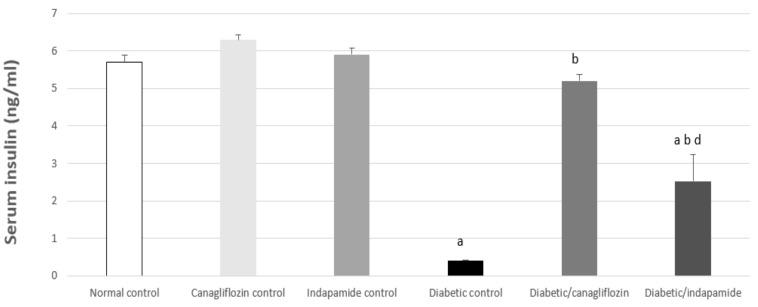
Impact of daily oral CANA and INDA treatment on serum insulin: oral CANA and INDA treatments were initiated once daily for 4 weeks. Data represent the mean ± S.E.M. (6 rats/group). ANOVA followed by post hoc Tukey–Kramer test at (*p* < 0.05) was used for statistical comparison; ^a^ against normal CTRL, ^b^ against diabetic CTRL, ^d^ against diabetic/CANA.

In context, there was a significant reduction of approximately 93% in serum insulin in the diabetic CTRL group (*p* < 0.05) compared to normal CTRL. Oral CANA (10 mg/kg) and INDA (10 mg/kg) treatment for 4 weeks significantly improved serum insulin levels, which exhibited a significant increase of approximately 13- (*p* < 0.05) and 6.3-fold (*p* < 0.05), respectively, compared to diabetic CTRL. CANA (10 mg/kg) was found to have a superior effect in terms of enhancing serum insulin levels (Figure 4), as it almost restored serum insulin levels to normal, on the basis of a comparison with the normal CTRL.

### 3.5. Impact of Daily CANA and INDA Treatment on HOMA-β

**Figure 5:**
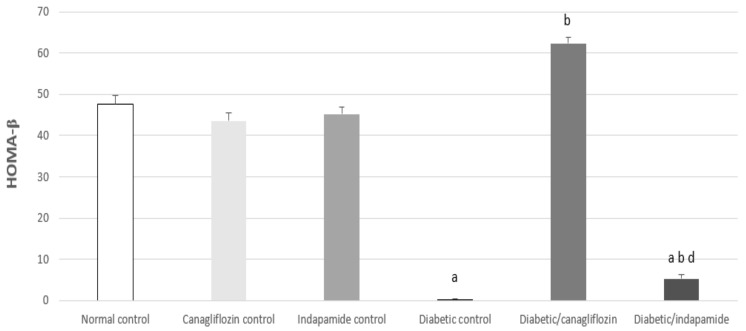
Impact of daily CANA and INDA treatment on HOMA-β: oral CANA and INDA treatments were initiated once daily for 4 weeks. Data represent the mean ± S.E.M. (6 rats/group). ANOVA followed by post hoc Tukey–Kramer test at (*p* < 0.05) was used for statistical comparison; ^a^ against normal CTRL, ^b^ against diabetic CTRL, ^d^ against diabetic/CANA.

The HOMA-β index is believed to be a significant measure for pancreatic β-cell functionality. It is evident from the results that the insulin secretory capacity of pancreatic β-cells in the diabetic CTRL was significantly diminished, compared to that of the normal CTRL, by about 99% (*p* < 0.05). Oral CANA (10 mg/kg) and INDA (10 mg/kg) treatment for 4 weeks significantly improved the secretory abilities of β-cells, with insulin secretion exhibiting significant increases of about 156- (*p* < 0.05) and 13-fold (*p* < 0.05), respectively, compared to the diabetic CTRL. CANA (10 mg/kg) had a superior effect in terms of improving β-cell secretory function compared to that of INDA, with glycemic control almost being restored to normal, on the basis of a comparison with the normal CTRL, Figure 5.

### 3.6. Impact of Daily CANA and INDA Treatment on Serum Adiponectin

**Figure 6:**
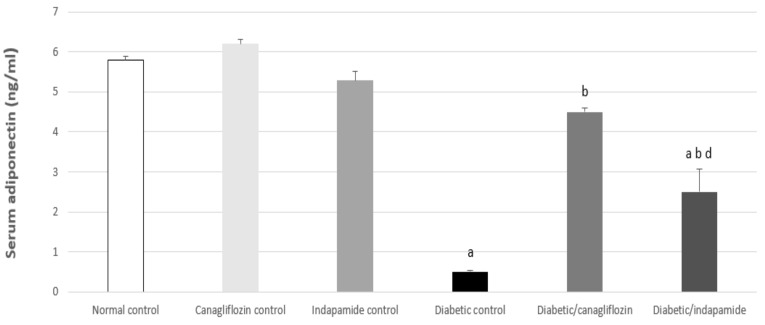
Impact of daily CANA and INDA treatment on serum adiponectin: oral CANA and INDA treatments were initiated once daily for 4 weeks. Data represent the mean ± S.E.M. (6 rats/group). ANOVA followed by post hoc Tukey–Kramer test at (*p* < 0.05) was used for statistical comparison; ^a^ against normal CTRL, ^b^ against diabetic CTRL, ^d^ against diabetic/CANA.

Diabetic CTRL showed a significant decrease in serum adiponectin concentration of approximately 91% (*p* < 0.05) compared to the normal CTRL. Oral CANA (10 mg/kg) and INDA (10 mg/kg) treatment for 4 weeks resulted in significant elevations in serum adiponectin by approximately 9- (*p* < 0.05) and 5-fold (*p* < 0.05), respectively, compared to the diabetic CTRL. CANA (10 mg/kg) boosted serum adiponectin levels more effectively than INDA. There was no significant difference between serum adiponectin for treatment with CANA and the normal CTRL (Figure 6).

### 3.7. Impact of Daily CANA and INDA Treatment on the TAC and MDA Contents in Epididymal Adipose Tissue and the Soleus Muscle

**Figure 7:**
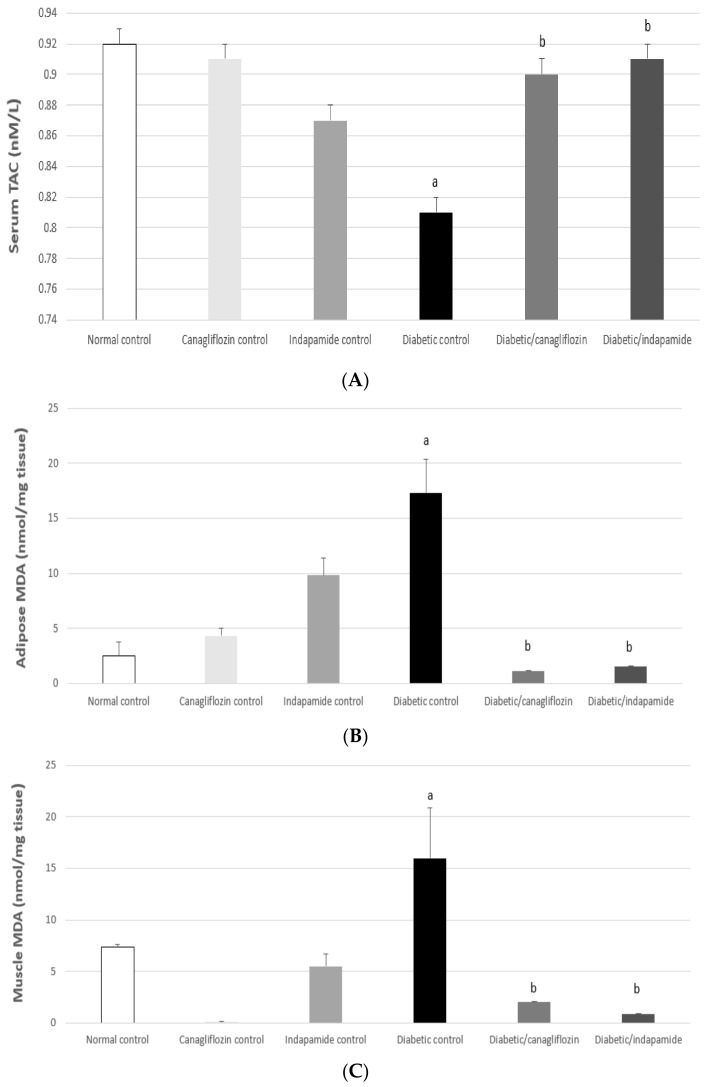
Impact of daily CANA and INDA treatment on serum TAC (**A**) and MDA content in epididymal adipose tissue (**B**) and soleus muscle (**C**) oral CANA and INDA treatments were initiated once daily for 4 weeks. Data represent the mean ± S.E.M. (6 rats/group). ANOVA followed by post hoc Tukey–Kramer test at (*p* < 0.05) was used for statistical comparison; ^a^ against normal CTRL, ^b^ against diabetic CTRL.

MDA—the product of lipid peroxidation—and TAC capacity represent sensors that are sensitive to the oxidative status within cells. In context, serum TAC exhibited a significant decrease of about 12% (*p* < 0.05) in the diabetic CTRL compared to the normal CTRL. Oral CANA (10 mg/kg) and INDA (10 mg/kg) treatment for 4 weeks resulted in a significant approximately 1.1-fold (*p* < 0.05) increase in serum TAC compared to the diabetic CTRL, while no significant difference was detected compared to normal CTRL. MDA contents in both epididymal adipose tissue and the soleus skeletal muscle in the diabetic CTRL rats exhibited significant increases of about 6.8- (*p* < 0.05) and 2.1-fold (*p* < 0.05), respectively, compared to normal CTRL. Oral CANA (10 mg/kg) and INDA (10 mg/kg) treatment for 4 weeks caused significant decreases in the MDA content of epididymal adipose tissue by approximately 94% (*p* < 0.05) and 91% (*p* < 0.05), respectively, and in the soleus muscle by approximately 88% (*p* < 0.05) and 95% (*p* < 0.05), respectively, compared to the diabetic CTRL, with no significant difference when compared to the normal CTRL (Figure 7).

### 3.8. Impact of Daily CANA and INDA Treatment on Nrf2 Content in Epididymal Adipose Tissue

**Figure 8:**
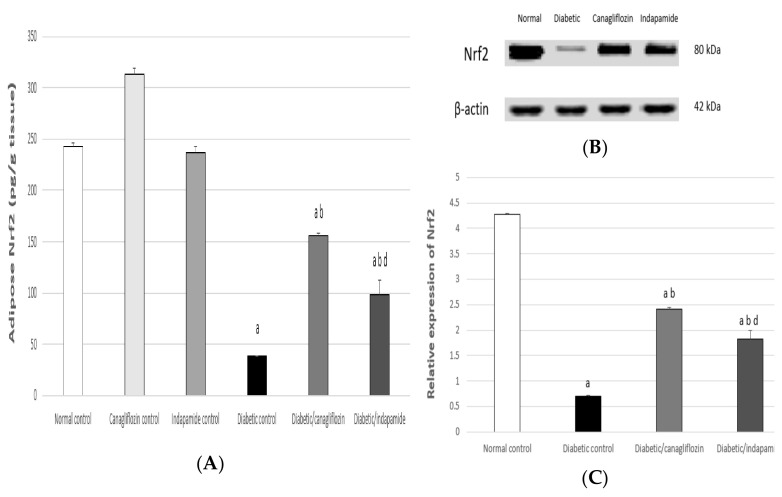
Impact of daily CANA and INDA treatment on epididymal adipose tissue Nrf2 content (ELISA) (**A**), (Western blotting) (**B**,**C**) oral CANA and INDA treatments were initiated once daily for 4 weeks. Data represent the mean ± S.E.M. (6 rats/group). ANOVA followed by post hoc Tukey–Kramer test at (*p* < 0.05) was used for statistical comparison; ^a^ against normal CTRL, ^b^ against diabetic CTRL, ^d^ against diabetic/CANA.

The Nrf2 content in epididymal adipose tissue exhibited a significant decrease of approximately 84% (*p* < 0.05) compared to the normal CTRL. Oral CANA (10 mg/kg) and INDA (10 mg/kg) treatment for 4 weeks significantly enhanced Nrf2 expression by approximately 4- (*p* < 0.05) and 3-fold (*p* < 0.05), respectively, compared to the diabetic CTRL. CANA (10 mg/kg) had a superior effect in terms of boosting Nrf2 expression in epididymal adipose tissue to that of INDA, but without significant difference compared to the normal CTRL (Figure 8).

### 3.9. Impact of Daily CANA and INDA Treatment on Serum TNF-α and IL-10

**Figure 9:**
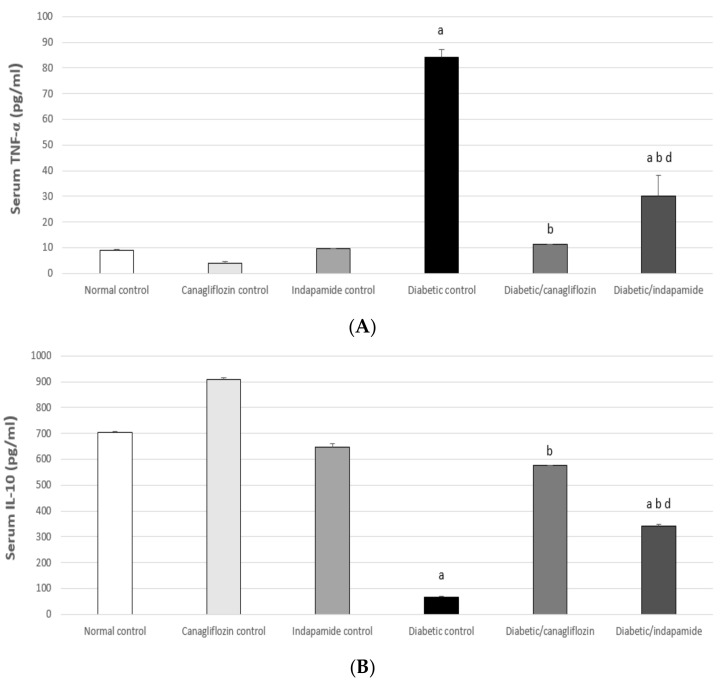
Impact of daily CANA and INDA treatment on serum TNF-α (**A**) and IL-10 (**B**) oral CANA and INDA treatments were initiated once daily for 4 weeks. Data represent the mean ± S.E.M. (6 rats/group). ANOVA followed by post hoc Tukey–Kramer test at (*p* < 0.05) was used for statistical comparison; ^a^ against normal CTRL, ^b^ against diabetic CTRL, ^d^ against diabetic/CANA.

Serum TNF-α was significantly increased in the diabetic CTRL rats by approximately 10-fold (*p* < 0.05) compared to the normal CTRL. Oral CANA (10 mg/kg) and INDA (10 mg/kg) treatment for 4 weeks caused significant decreases of approximately 87% (*p* < 0.05) and 64% (*p* < 0.05), respectively, in serum TNF-α compared to the diabetic CTRL. CANA (10 mg/kg) had a superior effect in terms of lowering serum TNF-α compared to INDA. Meanwhile, serum IL-10 significantly deteriorated in the diabetic CTRL, where it showed a significant decrease of approximately 90% (*p* < 0.05) compared to the normal CTRL. Oral CANA (10 mg/kg) and INDA (10 mg/kg) treatment for 4 weeks significantly enhanced serum IL-10, which exhibited approximately 9- (*p* < 0.05) and 5-fold (*p* < 0.05) increases, respectively, compared to the diabetic CTRL. CANA (10 mg/kg) had a superior impact on serum IL-10 concentration compared to INDA, but without significant difference compared to normal CTRL (Figure 9).

### 3.10. Impact of Daily CANA and INDA Treatment on NLRP3 Expression in Epididymal Adipose Tissue

**Figure 10:**
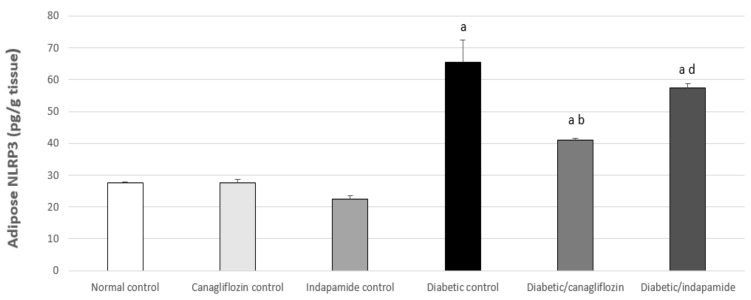
Impact of daily CANA and INDA treatment on epididymal adipose tissue NLRP3 expression: oral CANA and INDA treatments were initiated once daily for 4 weeks. Data represent the mean ± S.E.M. (6 rats/group). ANOVA followed by post hoc Tukey–Kramer test at (*p* < 0.05) was used for statistical comparison; ^a^ against normal CTRL, ^b^ against diabetic CTRL, ^d^ against diabetic/CANA.

Epididymal adipose NLRP3 expression was significantly increased in the diabetic CTRL by approximately 2.4-fold (*p* < 0.05) compared to the normal CTRL. Oral CANA (10 mg/kg) treatment for 4 weeks caused a significant decrease of approximately 37% (*p* < 0.05) in NLRP3 expression compared to the diabetic CTRL, but demonstrated no significant difference compared to the normal CTRL, while daily INDA (10 mg/kg, orally) did not induce a significant decrease in NLRP3 expression compared to the diabetic CTRL (Figure 10).

### 3.11. Impact of Daily CANA and INDA Treatment on Soleus Muscle IRS1 Expression

**Figure 11:**
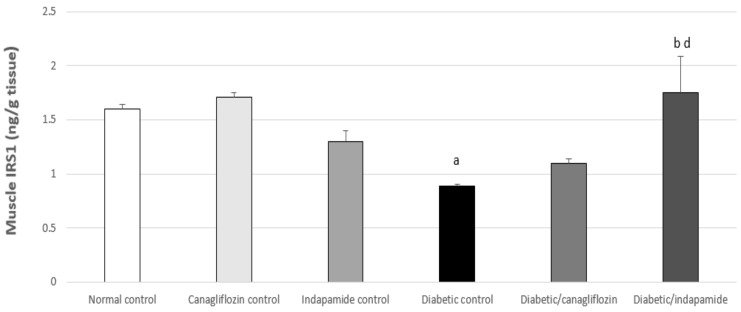
Impact of daily CANA and INDA treatment on soleus muscle IRS1 expression: oral CANA and INDA treatments were initiated once daily for 4 weeks. Data represent the mean ± S.E.M. (6 rats/group). ANOVA followed by post hoc Tukey–Kramer test at (*p* < 0.05) was used for statistical comparison; ^a^ against normal CTRL, ^b^ against diabetic CTRL, ^d^ against diabetic/CANA.

There was a significant decrease of about 44% (*p* < 0.05) in Soleus muscle IRS1 expression in the diabetic CTRL compared to the normal CTRL. Oral INDA (10 mg/kg) treatment for 4 weeks significantly boosted IRS1 expression about 2-fold (*p* < 0.05) compared to the diabetic CTRL. However, CANA (10 mg/kg) treatment did not induce a significant increase in IRS1 expression compared to the diabetic CTRL. Thus, in this aspect, INDA (10 mg/kg) had a superior effect in increasing IRS1 expression in the soleus muscle compared to that of CANA (Figure 11).

### 3.12. Impact of Daily CANA and INDA Treatment on PPARγ Expression in Epididymal Adipose Tissue

**Figure 12:**
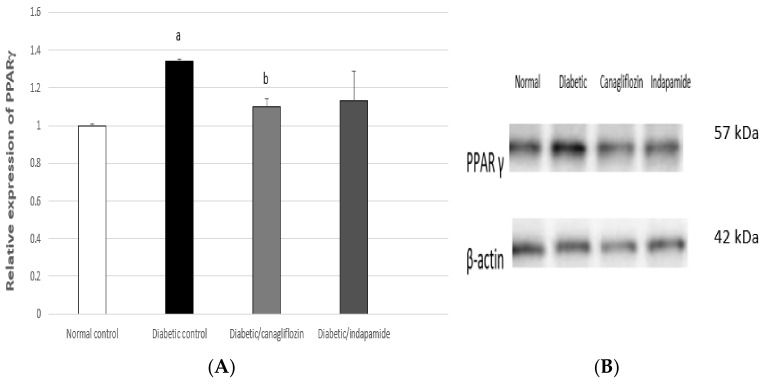
Impact of daily oral CANA and INDA treatment on epididymal adipose tissue (**A**) PPARγ expression: oral CANA and INDA treatments were initiated once daily for 4 weeks. (**B**) western blotting Data represent the mean ± S.E.M. (6 rats/group). ANOVA followed by post hoc Tukey–Kramer test at (*p* < 0.05) was used for statistical comparison; ^a^ against normal CTRL, ^b^ against diabetic CTRL.

The diabetic CTRL demonstrated a significant increase of approximately 34% (*p* < 0.05) in PPARγ expression in epididymal adipose tissue compared to the normal CTRL. Oral CANA (10 mg/kg) treatment for 4 weeks caused a significant decrease of approximately 18% (*p* < 0.05) in PPARγ expression compared to the diabetic CTRL, while oral INDA (10 mg/kg) did not induce significant suppression in PPARγ expression compared to the diabetic CTRL, exhibiting no significant difference compared to the normal CTRL (Figure 12).

### 3.13. Impact of Daily CANA and INDA Treatment on Morphological Changes in Epididymal Adipocytes

**Figure 13:**
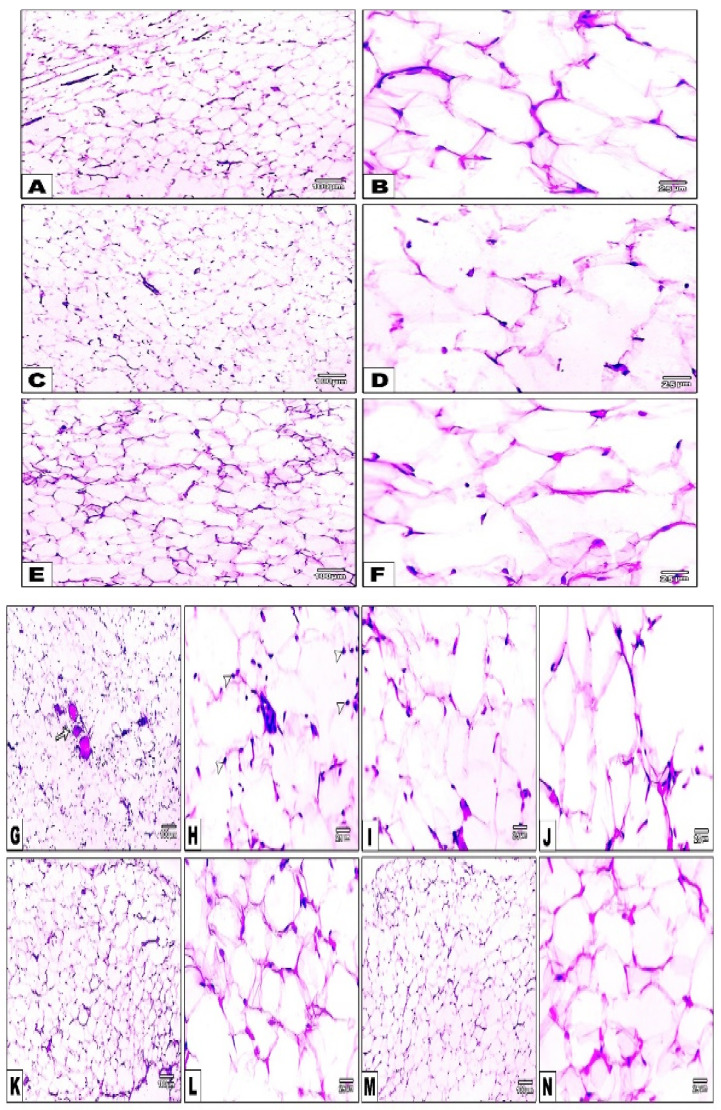

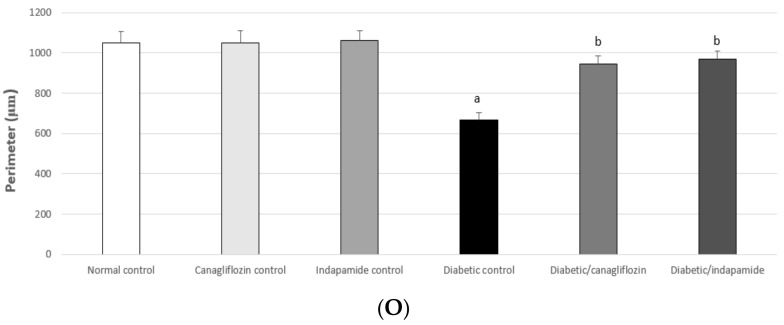
Adipose tissue specimens: normal (**A**,**B**), CANA (**C**,**D**), and INDA (**E**,**F**) CTRLs revealing the normal shape and size of the epididymal adipocytes; diabetic CTRL (**G**–**J**) showing congestion (arrow), decreased size of adipocytes, and macrophage infiltration (arrow heads). Marked increase in the size of the adipocytes in CANA (**K**,**L**) and INDA (**M**,**N**) treatment groups. Low magnification: 100× bar 100; high magnification: 400× bar 25. (**O**) Impact of daily treatment with CANA and INDA on epididymal adipocyte perimeter: oral CANA and INDA treatments were initiated once daily for 4 weeks. Data represent the mean ± S.E.M. (6 rats/group). ANOVA followed by post hoc Tukey–Kramer test at (*p* < 0.05) was used for statistical comparison; ^a^ against normal CTRL, ^b^ against diabetic CTRL.

The histopathological examination of the normal (Figure 13A,B), CANA (Figure 13C,D) and INDA (Figure 13E,F) CTRLs confirmed the normal shape and size of the adipocytes. The diabetic CTRL specimen (Figure 13G–J) exhibited a significant decline by approximately 36% (Figure 13 *p* < 0.05) in the perimeter of the epididymal adipocytes compared to the normal CTRL. Both CANA (10 mg/kg) (Figure 13K,L) and INDA (10 mg/kg) (Figure 13M,N) significantly enhanced the perimeter of the epididymal adipocytes, which exhibited a significant approximately 1.4-fold (*p* < 0.05) increase compared to the diabetic CTRL (Figure 13).

### 3.14. Impact of Daily CANA and INDA Treatment on Pancreatic Morphological Changes

**Figure 14:**
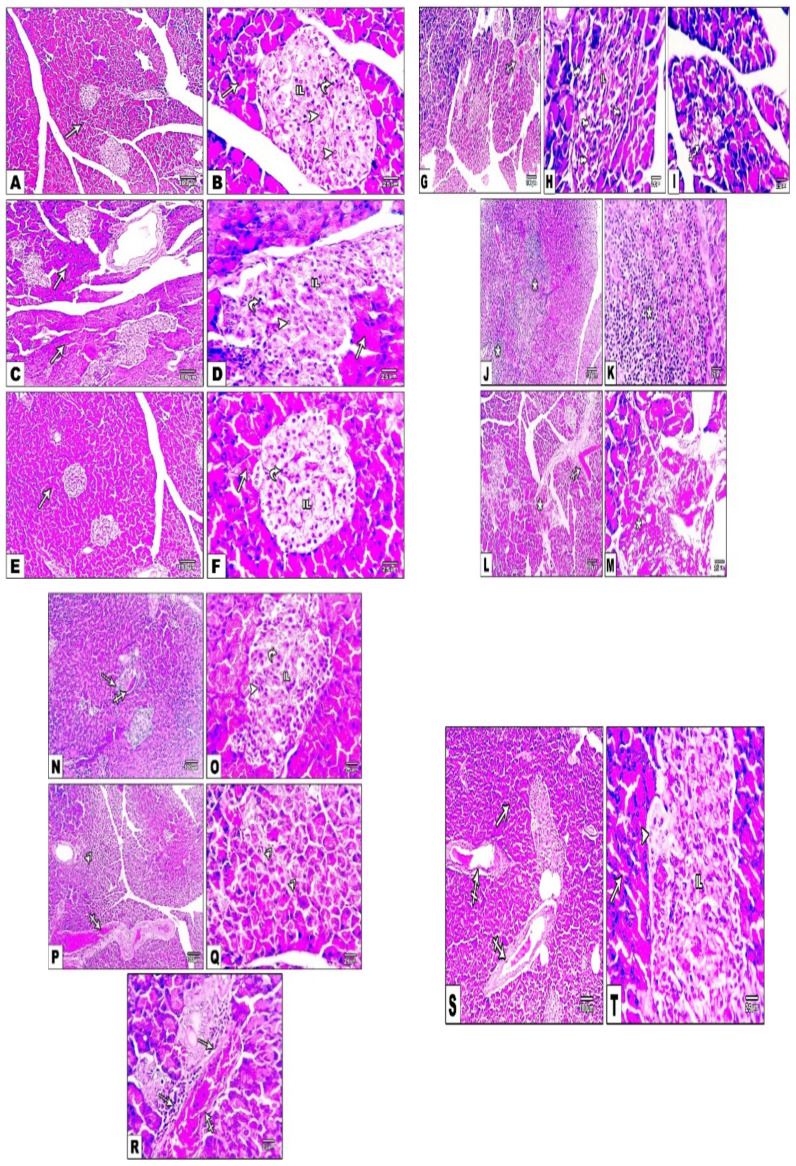
Pancreatic sections from normal (**A**,**B**), CANA (**C**,**D**) and INDA (**E**,**F**) CTRLs revealing well-preserved exocrine acini (arrows) and islets of Langerhans (IL); α (curved arrows) and β cells (arrowheads) in diabetic CTRL (**G**–**M**) showing congestion (crossed arrows), vacuolated exocrine acini (wavy arrow), and pyknotic nuclei in most α- and β-cells (rocket arrows) in the islets of the Langerhans (IL), confluent area of necrosis (stars), shrinkage of the pancreas, and vacuolation of β cells (short arrow). Pancreatic sections from the CANA-treated group (**N**–**R**) showing restored α (curved arrows) and β (arrowheads) cells, congestion (crossed arrows), focal damage to the exocrine acini (double-headed arrows) and infiltration of several perivascular mononuclear cells (dotted arrows). Pancreatic sections from the INDA-treated group (**S**,**T**) showing mild vacuolation of β cells (arrowheads), congestion (crossed arrows), and normal exocrine acini (arrows). Low magnification: 100× bar 100; high magnification: 400× bar 25.

Figure 14 presents sections from pancreatic specimens with normal structure of both the exocrine acini and the islets of Langerhans (IL); α- and β-cells in normal (Figure 14A,B), CANA (Figure 14C,D) and INDA (Figure 14E,F) CTRLs. The diabetic CTRL (Figure 14G–M) specimens exhibited significant loss of basophilia in the exocrine acini, and retraction in the size of the islets with vacuolation of the α- and β-cells. In the CANA-treated group (Figure 14N–R), restoration of α- and β-cells was revealed, along with congestion and focal injury of the exocrine acini with infiltration with few perivascular mononuclear cells. In the INDA-treated group (Figure 14S,T) revealed mildly vacuolated β-cells with mild congestion and normal exocrine acini.

### 3.15. Impact of Daily CANA and INDA Treatment on CD68 Expression in Epididymal Adipose Tissue

**Figure 15:**
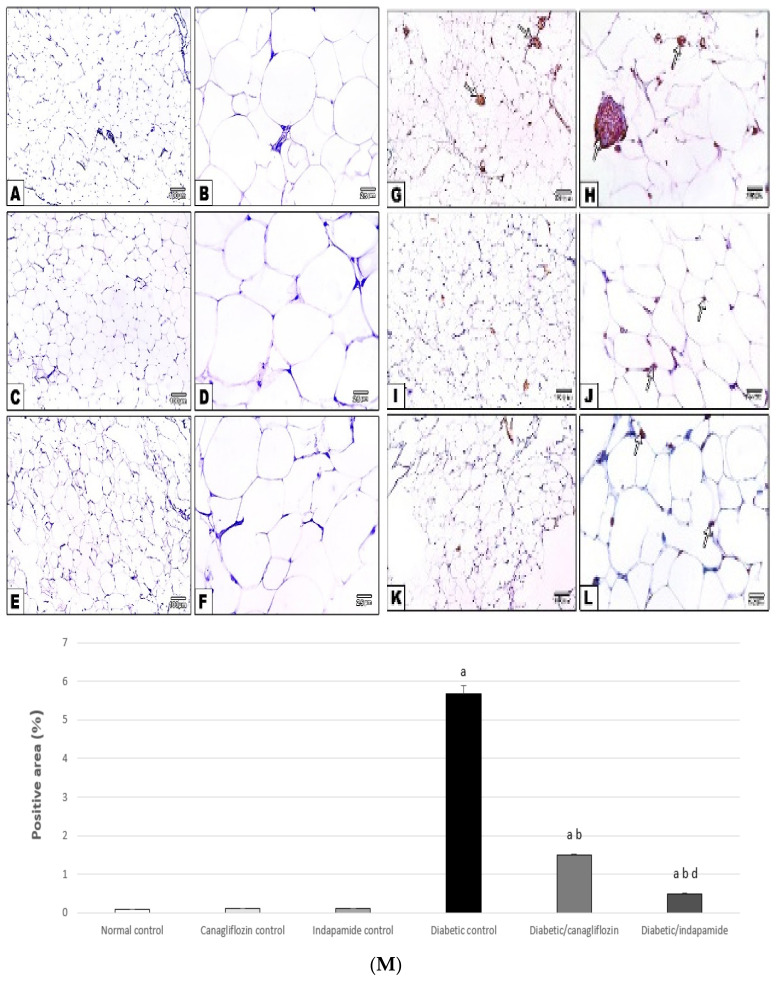
Immuno-stained adipose specimen against CD68 revealing negative immuno-staining in the normal (**A**,**B**), CANA (**C**,**D**), INDA (**E**,**F**) CTRLs. Increased positive immuno-staining of CD68-labeled macrophages (arrows) in the diabetic CTRL (**G**,**H**), and marked retraction in the CANA (**I**,**J**) and INDA (**K**,**L**) treatment groups. IHC counterstained with Mayer’s hematoxylin. Low magnification: 100× bar 100; high magnification: 400× bar 25. (**M**) Impact of daily treatment with CANA and INDA on CD68 expression in epididymal adipose tissue: oral CANA and INDA treatments were initiated once daily for 4 weeks. Data represent the mean ± S.E.M. (6 rats/group). ANOVA followed by post hoc Tukey–Kramer test at (*p* < 0.05) was used for statistical comparison; ^a^ against normal CTRL, ^b^ against diabetic CTRL, ^d^ against diabetic/CANA.

The percentage of CD68-positive immuno-stained area in the epididymal adipose tissue of the diabetic CTRL was significantly elevated by approximately 62-fold (*p* < 0.05) compared to the normal CTRL. Treatment with CANA (10 mg/kg) and INDA (10 mg/kg) for 4 weeks resulted in significant decreases of approximately 73% (*p* < 0.05) and 91% (*p* < 0.05), respectively, in CD68 expression compared to the diabetic CTRL. INDA (10 mg/kg) had a better effect in terms of reducing the expression of CD68 in epididymal adipose tissue (Figure 15).

### 3.16. Impact of Daily CANA and INDA Treatment on NF-κB Expression in Epididymal Adipose Tissue

**Figure 16:**
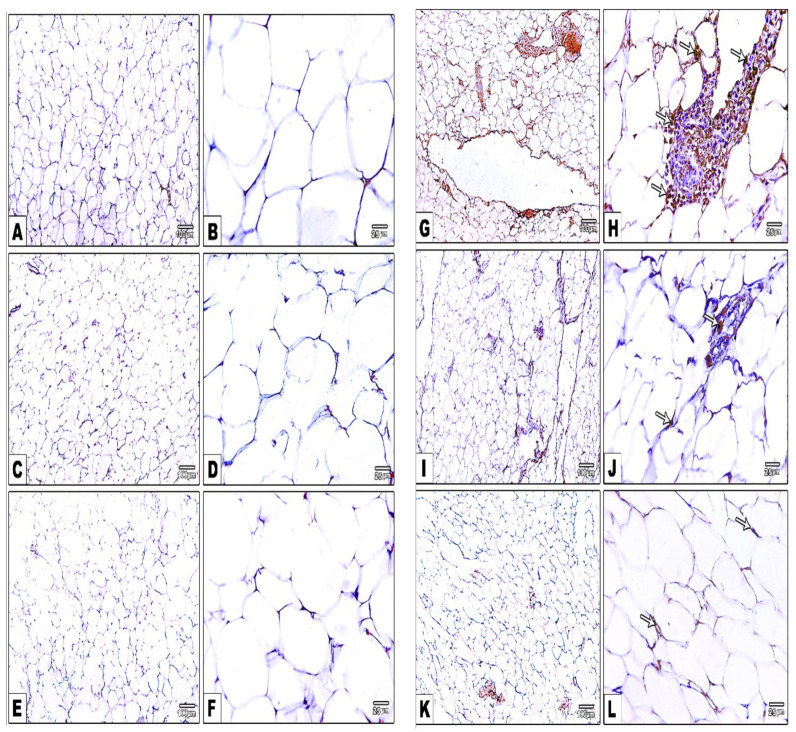

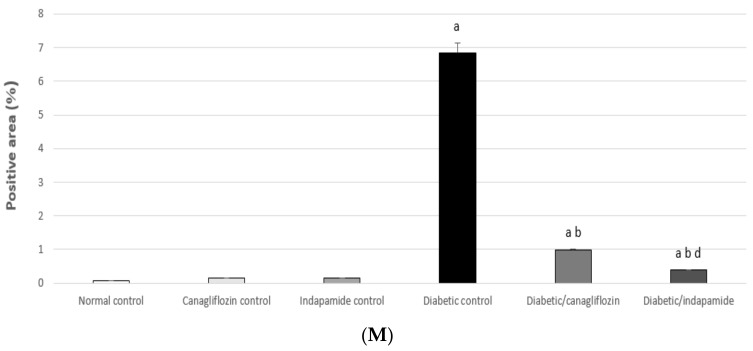
Immunostained epididymal adipose sections against NF-κB, confirming negative reaction and staining in the normal (**A**,**B**), CANA (**C**,**D**), and INDA (**E**,**F**) CTRLs. Enhanced positive NF-κB expression in diabetic CTRL (arrows) (**G**,**H**), and significantly decreased NF-κB expression in CANA (**I**,**J**) and INDA (**K**,**L**) treatment groups. IHC counterstained with Mayer’s hematoxylin. Low magnification: 100× bar 100; high magnification: 400× bar 25. (**M**) Impact of daily CANA and INDA treatment on NF-κB expression in the epididymal adipose tissue: oral CANA and INDA treatments were initiated once daily for 4 weeks. Data represent the mean ± S.E.M. (6 rats/group). ANOVA followed by post hoc Tukey–Kramer test at (*p* < 0.05) was used for statistical comparison; ^a^ against normal CTRL, ^b^ against diabetic CTRL, ^d^ against diabetic/CANA.

The percentage of NF-κB-positive immuno-stained area in the epididymal adipose tissue of the diabetic CTRL significantly increased by approximately 102-fold (*p* < 0.05) compared to the normal CTRL. Treatment with CANA (10 mg/kg) and INDA (10 mg/kg) for 4 weeks caused significant decreases of approximately 85% (*p* < 0.05) and 94% (*p* < 0.05), respectively, in NF-κB expression compared to the diabetic CTRL. INDA (10 mg/kg) was superior with respect to suppressing of NF-κB expression to INDA (Figure 16).

## 4. Discussion

T2DM is a multifactorial metabolic disorder that primarily affects glucose hemostasis in the human body. The main clinical and diagnostic features of T2DM are impaired glucose tolerance and hyperglycemia, and these are both also the direct result of either absolute or relative insulin deficiency or insulin resistance [20].

Adiponectin is a prominent adipocyte-secreted protein, and it is usually down-regulated in individuals suffering from obesity and its complications [21]. Unlike the other adipokines, adiponectin has been reported to demonstrate anti-diabetic, anti-inflammatory and anti-atherogenic properties [22]. Several approaches for maximizing adiponectin’s beneficial therapeutic impacts have been investigated, including increasing either its plasma level or its activity. Exogenously administered adiponectin, can also be used to elevate the circulating levels of adiponectin, and endogenous adiponectin’s impacts can be maximized indirectly through drugs that modulate either its expression or its functions. However, the administration of exogenous adiponectin represents a challenge due to its elevated endogenous circulating levels and its multimeric conformations. Hence, boosting endogenous adiponectin’s impacts using pharmacological agents, nutraceuticals, and lifestyle modification remains the best option [23].

The current study was conducted to evaluate the potential modulatory impact of CANA and INDA on endogenous adiponectin and to pinpoint the molecular pathways of the anti-diabetic effects of both drugs via adiponectin modulation in a rat model of T2DM. Thus, T2DM was established experimentally in rats using the fat plus fructose/STZ method [16,24].

Diabetic rats in the current study did not gain weight. This is consistent with Dai and Mcneill, 1995 [25], who reported that supplying the rats with 10% fructose caused a decline in diet consumption and failure to gain weight. Nevertheless, no weight gain was detected after 4–8 weeks of high fructose consumption [26]. The adjustment of the rats to the additional calories due to the consumption of 60% fructose resulted in the consumption of fewer calories from the diet, and thus the maintenance of normal B.W., which accounts for the observed retraction in B.W. gain with the consumption of the HFD [27].

Moreover, STZ-induced injury and subsequent destruction of β-cells led to an extensive retraction in serum insulin concentration. Insulin is an anabolic hormone with a stimulatory effect on protein metabolism, enhancing protein synthesis and decreasing its degradation. In turn, insulin insufficiency prompts the accelerated catabolism of proteins. Taken together, the previous explanation may account for the obvious decrease in B.W., which can primarily be attributed to inadequate insulin action. When this occurs, the body begins consuming fat and muscles instead for energy, causing a decrease in overall B.W. [28,29].

Treatment with CANA slightly reduced the rats’ B.W., but non-significantly, while treatment with INDA was associated with a significant loss in B.W. compared with both the initial rats’ B.W. and the diabetic CTRL. The observed reduction in B.W. with INDA therapy can be attributed to its diuretic properties.

As observed in the results of the present study, diabetic rats had the classical features of established DM, including significantly increased fasting blood glucose, HbA1c%, low serum insulin and HOMA-β. In T2DM, and despite the basal levels of β-cell’s regeneration and replication in the islets, β-cell apoptosis was enhanced, with an approximately 50% reduction in their number [30]. As T2DM progresses, the insulin-resistant status and the ensuing insulin deficiency forces a compensatory response in the β-cells to maintain normal blood glucose. Eventually, β-cell exhaustion prevails, with persistence of the hyperglycemic state in severely diabetic patients [31]. Nevertheless, the chronic persistence of high blood-free fatty acids induces β-cells lipotoxicity and suppresses their insulin secretory properties [32].

CANA and INDA significantly reduced HbA1c% and final blood glucose levels. These observations are in accordance with the reports of Sharabi et al., 1996 [33] and Henry et al., 2015 [34]. This hypoglycemic effect was further confirmed by the restoration of normal HOMA-β and insulin levels with CANA and INDA therapy. CANA and INDA might induce such recovery by means of the potentiation of pancreatic insulin secretion from the existing the β-cells as reported by Huang et al., 2009 [35]; Yang et al., 2020 [36].

CANA increased serum adiponectin levels, which is in line with the results of Garvey et al., 2018 [37]. Even though various studies have reported INDA to lower serum adiponectin levels [38,39], the current results confirm that INDA increases serum adiponectin levels.

Oxidative stress, which develops with T2DM secondary to the elevation in glucose and/or fatty acid oxidation, induces uncurbed production of reactive oxygen species (ROS), which further suppresses insulin release, negatively impacts insulin sensitivity, and interferes with insulin signaling in insulin-responsive tissues. Indeed, increased intracellular ROS production following mitochondrial dysfunction has been reported to compromise adipocyte functions and suppress adiponectin secretion [40]. Previous reports have confirmed a positive correlation between both adiponectin and the biomarkers of the antioxidant elements, including TAC [41,42,43], and the current results confirm this. Furthermore, a negative correlation was between MDA and adiponectin level in the current study as well, which is also in agreement with the observations of [44].

Both CANA and INDA reduced MDA content in both the adipocytes and the muscles, which is in agreement with the observations of Ma et al., 2013 [45] and Hasan et al., 2020 [46]. Moreover, both drugs significantly boosted serum TAC, which is consistent with the observations of Kojsová et al., 2006 [47] and Kabil and Mahmoud, 2018 [48].

Nrf2 is a transcription factor that regulates the cellular antioxidant response by enhancing the expression of endogenous antioxidant and cytoprotective enzymes [49]. Adiponectin has been reported to ameliorate cardiac hypertrophy by preserving myocardial Nrf2 expression [50]. Moreover, Hasan et al., 2020 [46] and Schragenheim et al., 2018 [51] confirmed CANA and INDA to enhance Nrf2 expression in epididymal adipose tissue, with significant decreases in oxidative load and lipid peroxidation, and enhancement of the TAC, thus confirming their antioxidant impact.

Inflammasome activation and ROS production have been reported to be strongly associated. Different signaling pathways are thought to be integrated by ROS, resulting in their production and inflammasome activation [52]. Upon proinflammatory stimulation, the NLRP3 inflammasome, a significant inflammatory mediator, is assembled. Moreover, NF-κB signaling is crucial for proper NLRP3 inflammasome activation. Interestingly, hyperglycemia has been reported to activate NLRP3 inflammasomes and downstream effector inflammatory cytokines, including TNF-α and IL-1β [53], which are believed to induce programmed proinflammatory cell death (pyroptosis) [54].

In agreement with Ma et al., 2013 [45], Kabil and Mahmoud, 2018 [48], Inoue et al., 2019 [55] and Brown et al., 2021 [56], both CANA and INDA down-regulated the inflammatory pathway mediated by NF-κB and NLRP3. On the other hand, the results of Morsy et al., 2021 [57] were along the same lines as the current observations regarding the enhancing impact of CANA on serum levels of IL-10. To the best of our knowledge, no previously published literature has discussed the impact of INDA on IL-10.

The binding of adiponectin to AdipoR1 would fully repress NF-κB signaling and cause a decrease in muscle inflammation. Moreover, adiponectin has been reported to enhance insulin sensitivity and glucose uptake by prompting IRS1 binding to its insulin receptor [58]. In context, CANA and INDA enhanced the expression of IRS1 in the soleus muscle in the current study.

White and brown adipose tissue express PPARγ in high concentrations, where it is believed to be involved in the mediation of numerous biological processes, including adipogenesis, glucose homeostasis, atherogenesis, inflammation, and tumor susceptibility [59]. HFD consumption has been reported to be associated with a significant reduction in PPARγ expression in adipocytes [60,61]. On the other hand, Ji et al., 2017 [62] and Sikder et al., 2018 [63] reported HFD to markedly enhance PPARγ expression, which confirms the results observed in the current study. CANA caused a significant decrease in PPARγ expression, which is also in agreement with the observations of Ji et al., 2017 [62]. To the best of our knowledge, no previously published literature has discussed the impact of INDA on PPARγ.

The use of HFD to induce T2DM is associated with the hypertrophy of adipocytes due to enhanced energy intake [64,65]. The incorporation of STZ to HFD for the establishment of T2DM is in line with the reports of Jung et al., 2017 [66] and Yin et al., 2020 [67]. The lack of insulin accelerates lipolysis that accompanies the uncontrolled diabetic state may account for the reduction in adipocyte perimeter. On the other hand, the results observed in the current study confirm that CANA and INDA treatments are able to restore the size of epididymal adipocytes.

CANA attenuated the morphological alterations in the islets, which is in accordance with the observations of Mohamed et al., 2021 [68], and this was also consistent with the biochemical enhancements, and, notably, the effect of CANA on these parameters was significantly superior to that of INDA, which might explain the superior anti-inflammatory and antioxidant effects observed for CANA compared to those observed for INDA.

In agreement with Brown et al., 2021 [56] and Nasiri-Ansari et al., 2018 [69], both CANA and INDA suppressed the NF-κB-mediated signaling pathway via suppression of CD68 expression in epididymal adipose tissue, further confirming the modulatory impact of adiponectin on inflammatory signaling and confirming the anti-inflammatory properties of adiponectin, as it reduces inflammation by targeting the differentiation and function of macrophages. It suppresses differentiation of myeloid progenitor cells, inhibits foam cells formation from macrophages, promotes macrophages polarization to an M2 anti-inflammatory state, and decreases the expression of toll-like receptor 4 (TLR4) in macrophages and progenitor cells [70].

## 5. Conclusions

CANA and INDA improved DM indices with an underlined ability to enhance insulin secretion, and modulate and preserve adiponecting concentration, in parallel with a significant preservation of the histopathogical characteristics of both adipocytes and pancreatic β-cells. Meanwhile, glucose use in the skeletal muscles significantly improved. Both CANA and INDA targeted key intracellular mediators empirically linked to the pathogenesis of T2DM and its related pathologies, including inflammation, oxidative stress, infiltration of adipocytes by macrophages, and insulin use in the skeletal muscles. Of note, the effects of CANA and INDA were comparable on the inflammatory and antioxidant scales. Nevertheless, CANA was superior to INDA as a modulator of adiponectin secretion and insulin secretion, and possessed a prominent anti-inflammatory impact.

## Data Availability

Data is contained within the article.

## References

[1] Samadi N., Mozaffari-Khosravi H., Rahmanian M., Askarishahi M. (2017). Effects of bee propolis supplementation on glycemic control, lipid profile and insulin resistance indices in patients with type 2 diabetes: A randomized, double-blind clinical trial. J. Integr. Med..

[2] Al-Assaf A.H. (2012). Antihyperglycemic and antioxidant effect of ginger extract on streptozotocin-diabetic rats. Pak. J. Nutr..

[3] Forbes J.M., Cooper M.E. (2013). Mechanisms of diabetic complications. Physiol. Rev..

[4] Lindberg S., Jensen J.S., Bjerre M., Pedersen S.H., Frystyk J., Flyvbjerg A., Galatius S., Jeppesen J., Mogelvang R. (2015). Adiponectin, type 2 diabetes and cardiovascular risk. Eur. J. Prev. Cardiol..

[5] Kadowaki T., Yamauchi T., Kubota N., Hara K., Ueki K., Tobe K. (2006). Adiponectin and adiponectin receptors in insulin resistance, diabetes, and the metabolic syndrome. J. Clin. Investig..

[6] Guo R., Zhang Y., Turdi S., Ren J. (2013). Adiponectin knockout accentuates high fat diet-induced obesity and cardiac dysfunction: Role of autophagy. Biochim. Biophys. Acta.

[7] Lee B., Shao J. (2012). Adiponectin and lipid metabolism in skeletal muscle. Acta Pharm. Sin. B.

[8] Xu A., Wang H., Hoo R.L., Sweeney G., Vanhoutte P.M., Wang Y., Wu D., Chu W., Qin G., Lam K.S. (2009). Selective elevation of adiponectin production by the natural compounds derived from a medicinal herb alleviates insulin resistance and glucose intolerance in obese mice. Endocrinology.

[9] Meng X.M., Ma X.X., Tian Y.L., Jiang Q., Wang L.L., Shi R., Ding L., Pang S.G. (2017). Metformin improves the glucose and lipid metabolism via influencing the level of serum total bile acids in rats with streptozotocin-induced type 2 diabetes mellitus. Eur. Rev. Med. Pharmacol. Sci..

[10] Gabr N.M., Mohammed I.H. (2020). A comparative study of canagliflozin (INVOKANA) on type-I and type-II diabetes mellitus on adult male albino rat. Al-Azhar Med. J..

[11] Sassard J., Bataillard A., McIntyre H. (2005). An overview of the pharmacology and clinical efficacy of indapamide sustained release. Fundam. Clin. Pharmacol..

[12] Kuo S.W., Hung Y.J., Hsieh A.T., Wu L.Y., Hsieh C.H., He C.T., Yang T.C., Lian W.C. (2003). Effect of indapamide SR in the treatment of hypertensive patients with type 2 diabetes. Am. J. Hypertes..

[13] Samaha M.M., Said E., Salem H.A. (2019). A comparative study of the role of crocin and sitagliptin in attenuation of STZ-induced diabetes mellitus and the associated inflammatory and apoptotic changes in pancreatic β-islets. Environ. Toxicol. Pharmacol..

[14] Luo C., Yang H., Tang C., Yao G., Kong L., He H., Zhou Y. (2015). Kaempferol alleviates insulin resistance via hepatic IKK/NF-κB signal in type 2 diabetic rats. Int. Immunopharmacol..

[15] Kumar S.A., Magnusson M., Ward L.C., Paul N.A., Brown L. (2015). Seaweed supplements normalise metabolic, cardiovascular and liver responses in high- carbohydrate, high-fat fed rats. Mar. Drugs.

[16] Samaha M.M., Said E., Salem H.A. (2020). Modulatory role of imatinib mesylate on pancreatic β-cells’ secretory functions in an STZ rat model of diabetes mellitus. Chem. Biol. Interact..

[17] Burnette W.N. (1981). “Western blotting”: Electrophoretic transfer of proteins from sodium dodecyl sulfate—polyacrylamide gels to unmodified nitrocellulose and radiographic detection with antibody and radioiodinated protein A. Anal. Biochem..

[18] Sambrook J., Fritsch E.F., Maniatis T. (1989). Molecular Cloning: A Laboratory Manual.

[19] Matthews D.R., Hosker J.P., Rudenski A.S., Naylor B.A., Treacher D.F., Turner R.C. (1985). Homeostasis model assessment: Insulin resistance and beta-cell function from fasting plasma glucose and insulin concentrations in man. Diabetologia.

[20] Inzucchi S.E. (2012). Diagnosis of diabetes. N. Engl. J. Med..

[21] Arita Y., Kihara S., Ouchi N., Takahashi M., Maeda K., Miyagawa J., Hotta K., Shimomura I., Nakamura T., Miyaoka K. (1999). Paradoxical decrease of an adipose-specific protein, adiponectin, in obesity. Biochem. Biophys. Res. Commun..

[22] Yadav A., Kataria M.A., Saini V., Yadav A. (2013). Role of leptin and adiponectin in insulin resistance. Clin. Chim. Acta.

[23] Caselli C. (2014). Role of adiponectin system in insulin resistance. Mol. Genet. Metab..

[24] Wong W.Y., Ward L.C., Fong C.W., Yap W.N., Brown L. (2017). Anti-inflammatory γ- and δ-tocotrienols improve cardiovascular, liver and metabolic function in diet-induced obese rats. Eur. J. Nutr..

[25] Dai S., McNeill J.H. (1995). Fructose-induced hypertension in rats is concentration- and duration-dependent. J. Pharmacol. Toxicol. Methods.

[26] Galipeau D., Arikawa E., Sekirov I., McNeill J.H. (2001). Chronic thromboxane synthase inhibition prevents fructose-induced hypertension. Hypertension.

[27] Stark A.H., Timar B., Madar Z. (2000). Adaptation of Sprague Dawley rats to long-term feeding of high fat or high fructose diets. Eur. J. Nutr..

[28] Ozougwu J.C., Obimba K.C., Belonwu C.D., Unakalamba C.B. (2013). The pathogenesis and pathophysiology of type 1 and type 2 diabetes mellitus. J. Physiol. Pathophysiol..

[29] Altinoz E., Oner Z., Elbe H., Cigremis Y., Turkoz Y. (2015). Protective effects of saffron (its active constituent, crocin) on nephropathy in streptozotocin-induced diabetic rats. Hum. Exp. Toxicol..

[30] Butler A.E., Janson J., Bonner-Weir S., Ritzel R., Rizza R.A., Butler P.C. (2003). Beta- cell deficit and increased beta-cell apoptosis in humans with type 2 diabetes. Diabetes.

[31] Robertson R.P., Harmon J., Tran P.O., Tanaka Y., Takahashi H. (2003). Glucose toxicity in beta-cells: Type 2 diabetes, good radicals gone bad, and the glutathione connection. Diabetes.

[32] Oh Y.S., Bae G.D., Baek D.J., Park E.Y., Jun H.S. (2018). Fatty Acid-Induced Lipotoxicity in Pancreatic Beta-Cells During Development of Type 2 Diabetes. Front. Endocrinol..

[33] Sharabi Y., Grossman E., Nussinovitch N., Katz A., Rachima-Moaz C., Rosenthal T. (1996). Indapamide—A substitute diuretic for hypertensives with hyperglycemia and/or dyslipidemia. Harefuah.

[34] Henry R.R., Thakkar P., Tong C., Polidori D., Alba M. (2015). Efficacy and Safety of Canagliflozin, a Sodium-Glucose Cotransporter 2 Inhibitor, as Add-on to Insulin in Patients with Type 1 Diabetes. Diabetes Care.

[35] Huang S.S., Wu T.C., Lin S.J., Chen J.W. (2009). Combination of an ACE inhibitor and indapamide improves blood pressure control, but attenuates the beneficial effects of ACE inhibition on plasma adiponectin in patients with essential hypertension. Circ. J..

[36] Yang Y., Zhao C., Ye Y., Yu M., Qu X. (2020). Prospect of Sodium-Glucose Co-transporter 2 Inhibitors Combined with Insulin for the Treatment of Type 2 Diabetes. Front. Endocrinol..

[37] Garvey W.T., Van Gaal L., Leiter L.A., Vijapurkar U., List J., Cuddihy R., Ren J., Davies M.J. (2018). Effects of canagliflozin versus glimepiride on adipokines and inflammatory biomarkers in type 2 diabetes. Metabolism.

[38] Piecha G., Adamczak M., Chudek J., Wiecek A. (2007). Indapamide decreases plasma adiponectin concentration in patients with essential hypertension. Kidney Blood Press. Res..

[39] Brody R., Peleg E., Grossman E., Sharabi Y. (2009). Production and secretion of adiponectin from 3T3-L1 adipocytes: Comparison of antihypertensive drugs. Am. J. Hypertens..

[40] Wang C.H., Wang C.C., Huang H.C., Wei Y.H. (2013). Mitochondrial dysfunction leads to impairment of insulin sensitivity and adiponectin secretion in adipocytes. FEBS J..

[41] Nakanishi S., Yamane K., Kamei N., Nojima H., Okubo M., Kohno N. (2005). A protective effect of adiponectin against oxidative stress in Japanese Americans: The association between adiponectin or leptin and urinary isoprostane. Metabolism.

[42] Kaur S., Zilmer K., Kairane C., Kals M., Zilmer M. (2008). Clear differences in adiponectin level and glutathione redox status revealed in obese and normal-weight patients with psoriasis. Br. J. Dermatol..

[43] Li J., Shen X. (2019). Oxidative stress and adipokine levels were significantly correlated in diabetic patients with hyperglycemic crises. Diabetol. Metab. Syndr..

[44] Yang W.S., Lee W.J., Funahashi T., Tanaka S., Matsuzawa Y., Chao C.L., Chen C.L., Tai T.Y., Chuang L.M. (2001). Weight reduction increases plasma levels of an adipose- derived anti-inflammatory protein, adiponectin. J. Clin. Endocrinol. Metab..

[45] Ma F., Lin F., Chen C., Cheng J., Zeldin D.C., Wang Y., Wang D.W. (2013). Indapamide lowers blood pressure by increasing production of epoxyeicosatrienoic acids in the kidney. Mol. Pharmacol..

[46] Hasan R., Lasker S., Hasan A., Zerin F., Zamila M., Parvez F., Rahman M.M., Khan F., Subhan N., Alam M.A. (2020). Canagliflozin ameliorates renal oxidative stress and inflammation by stimulating AMPK-Akt-eNOS pathway in the isoprenaline-induced oxidative stress model. Sci. Rep..

[47] Kojsová S., Jendeková L., Zicha J., Kunes J., Andriantsitohaina R., Pechánová O. (2006). The effect of different antioxidants on nitric oxide production in hypertensive rats. Physiol. Res..

[48] Kabil S.L., Mahmoud N.M. (2018). Canagliflozin protects against non-alcoholic steatohepatitis in type-2 diabetic rats through zinc alpha-2 glycoprotein up-regulation. Eur. J. Pharmacol..

[49] Elsherbiny N.M., Said E., Atef H., Zaitone S.A. (2020). Renoprotective effect of calycosin in high fat diet-fed/STZ injected rats: Effect on IL-33/ST2 signaling, oxidative stress and fibrosis suppression. Chem. Biol. Interact..

[50] Li H., Yao W., Irwin M.G., Wang T., Wang S., Zhang L., Xia Z. (2015). Adiponectin ameliorates hyperglycemia-induced cardiac hypertrophy and dysfunction by concomitantly activating Nrf2 and Brg1. Free Radic. Biol. Med..

[51] Schragenheim J., Bellner L., Cao J., Singh S.P., Bamshad D., McClung J.A., Maayan O., Meissner A., Grant I., Stier C.T. (2018). EET enhances renal function in obese mice resulting in restoration of HO-1-Mfn1/2 signaling, and decrease in hypertension through inhibition of sodium chloride co-transporter. Prostaglandins Other Lipid Mediat..

[52] Tschopp J., Schroder K. (2010). NLRP3 inflammasome activation: The convergence of multiple signalling pathways on ROS production?. Nat. Rev. Immunol..

[53] Alzahrani S., Zaitone S.A., Said E., El-Sherbiny M., Ajwah S., Alsharif S.Y., Elsherbiny N.M. (2020). Protective effect of isoliquiritigenin on experimental diabetic nephropathy in rats: Impact on Sirt-1/NFκB balance and NLRP3 expression. Int. Immunopharmacol..

[54] Mahmoud A.M., Hussein O.E., Abd El-Twab S.M., Hozayen W.G. (2019). Ferulic acid protects against methotrexate nephrotoxicity via activation of Nrf2/ARE/HO-1 signaling and PPARγ, and suppression of NF-κB/NLRP3 inflammasome axis. Food Funct..

[55] Inoue M.K., Matsunaga Y., Nakatsu Y., Yamamotoya T., Ueda K., Kushiyama A., Sakoda H., Fujishiro M., Ono H., Iwashita M. (2019). Possible involvement of normalized Pin1 expression level and AMPK activation in the molecular mechanisms underlying renal protective effects of SGLT2 inhibitors in mice. Diabetol. Metab. Syndr..

[56] Brown D., Moezzi D., Dong Y., Koch M., Yong V.W. (2021). Combination of Hydroxychloroquine and Indapamide Attenuates Neurodegeneration in Models Relevant to Multiple Sclerosis. Neurotherapeutics.

[57] Morsy M.A., Khalaf H.M., Rifaai R.A., Bayoumi A., Khalifa E., Ibrahim Y.F. (2021). Canagliflozin, an SGLT-2 inhibitor, ameliorates acetic acid-induced colitis in rats through targeting glucose metabolism and inhibiting NOX2. Biomed. Pharmacother..

[58] Abou-Samra M., Selvais C.M., Dubuisson N., Brichard S.M. (2020). Adiponectin and Its Mimics on Skeletal Muscle: Insulin Sensitizers, Fat Burners, Exercise Mimickers, Muscling Pills … or Everything Together?. Int. Mol. Sci..

[59] Jones J.R., Barrick C., Kim K.A., Lindner J., Blondeau B., Fujimoto Y., Shiota M., Kesterson R.A., Kahn B.B., Magnuson M.A. (2005). Deletion of PPARgamma in adipose tissues of mice protects against high fat diet-induced obesity and insulin resistance. Proc. Natl. Acad. Sci. USA.

[60] Eissa L.A., Elsherbiny N.M., Maghmomeh A.O. (2017). Effect of 2-hydroxychalcone on adiponectin level in type 2 diabetes induced experimentally in rats. Egypt. J. Basic Appl. Sci..

[61] Mahmoud A.M., Abdel-Rahman M.M., Bastawy N.A., Eissa H.M. (2017). Modulatory effect of berberine on adipose tissue PPARγ, adipocytokines and oxidative stress in high fat diet/streptozotocin-induced diabetic rats. J. Appl. Pharm. Sci..

[62] Ji W., Zhao M., Wang M., Yan W., Liu Y., Ren S., Lu J., Wang B., Chen L. (2017). Effects of canagliflozin on weight loss in high-fat diet-induced obese mice. PLoS ONE.

[63] Sikder K., Shukla S.K., Patel N., Singh H., Rafiq K. (2018). High Fat Diet Upregulates Fatty Acid Oxidation and Ketogenesis via Intervention of PPAR-γ. Cell. Physiol. Biochem..

[64] Gao M., Ma Y., Liu D. (2015). High-fat diet-induced adiposity, adipose inflammation, hepatic steatosis and hyperinsulinemia in outbred CD-1 mice. PLoS ONE.

[65] Gamil N.M., Abd El Fattah M.A., Ahmed M., Maklad Y.A., Gamal El Din A.A., Eid N.I. (2020). Lansoprazole enhances the antidiabetic effect of dapagliflozin in fortified diet- fed streptozotocin-treated diabetic rats. J. Biochem. Mol. Toxicol..

[66] Jung Y.J., Park W., Nguyen-Thanh T., Kang K.P., Jin H.Y., Kim S.H., Suh W., Kim W. (2017). COMP-angiopoietin-1 mitigates changes in lipid droplet size, macrophage infiltration of adipose tissue and renal inflammation in streptozotocin-induced diabetic mice. Onotarget.

[67] Yin R., Xue Y., Hu J., Hu X., Shen Q. (2020). The effects of diet and streptozotocin on metabolism and gut microbiota in a type 2 diabetes mellitus mouse model. Food Agric. Immunol..

[68] Mohamed T.Y.H., Ahmed M.A., Mahmoud S.S. (2021). Comparative Study on the Cardiovascular and Pancreatic Effects of Canagliflozin versus Vildagliptin on Experimentally Induced Diabetes and Hypertension in Male Albino Rats. Eur. J. Mol. Clin. Med..

[69] Nasiri-Ansari Ν., Dimitriadis G.K., Agrogiannis G., Perrea D., Kostakis I.D., Kaltsas G., Papavassiliou A.G., Randeva H.S., Kassi E. (2018). Canagliflozin attenuates the progression of atherosclerosis and inflammation process in APOE knockout mice. Cardiovasc. Diabetol..

[70] Fang H., Judd R.L. (2018). Adiponectin Regulation and Function. Compr. Physiol..

